# Effect of COVID-19 Pandemic on Patients Who Have Undergone Liver Transplantation: Retrospective Cohort Study

**DOI:** 10.3390/jcm12134466

**Published:** 2023-07-03

**Authors:** Sami Akbulut, Fatma Hilal Yagin, Tevfik Tolga Sahin, Ibrahim Umar Garzali, Adem Tuncer, Musap Akyuz, Nazlican Bagci, Bora Barut, Selver Unsal, Kemal Baris Sarici, Serdar Saritas, Ali Ozer, Recep Bentli, Cemil Colak, Yasar Bayindir, Sezai Yilmaz

**Affiliations:** 1 Department of Surgery and Liver Transplant Institute, Inonu University Faculty of Medicine, Malatya 44280, Turkey; 2 Department of Biostatistics and Medical Informatics, Inonu University Faculty of Medicine, Malatya 44280, Turkey; 3 Department of Public Health, Inonu University Faculty of Medicine, Malatya 44280, Turkey; 4 Department of Surgery, Aminu Kano Teaching Hospital, Kano 700101, Nigeria; 5 Department of Surgical Nursing, Inonu University Faculty of Nursing, Malatya 44280, Turkey; 6 Department of Nursing Service, Inonu University Faculty of Medicine, Malatya 44280, Turkey; 7 Department of Internal Medicine, Inonu University Faculty of Medicine, Malatya 44280, Turkey; 8 Department of Infectious Diseases and Clinical Microbiology, Inonu University Faculty of Medicine, Malatya 44280, Turkey

**Keywords:** COVID-19 pandemic, liver transplantation, immunosuppressive agents, COVID-19 vaccine

## Abstract

Background: In liver transplant (LT) recipients, immunosuppressive therapy may potentially increase the risk of severe COVID-19 and may increase the mortality in patients. However, studies have shown conflicting results, with various studies reporting poor outcomes while the others show no difference between the LT recipients and healthy population. The aim of this study is to determine the impact of the COVID-19 pandemic on survival of LT recipients. Methods: This is a retrospective cohort study analyzing the data from 387 LT recipients diagnosed with COVID-19. LT recipients were divided into two groups: survival (*n* = 359) and non-survival (*n* = 28) groups. A logistic regression model was used to determine the independent risk factors for mortality. Machine learning models were used to analyze the contribution of independent variables to the mortality in LT recipients. Results: The COVID-19-related mortality rate in LT recipients was 7.2%. Multivariate analysis showed that everolimus use (*p* = 0.012; OR = 6.2), need for intubation (*p* = 0.001; OR = 38.4) and discontinuation of immunosuppressive therapy (*p* = 0.047; OR = 7.3) were independent risk factors for mortality. Furthermore, COVID-19 vaccination reduced the risk of mortality by 100 fold and was the single independent factor determining the survival of the LT recipients. Conclusion: The effect of COVID-19 infection on LT recipients is slightly different from the effect of the disease on the general population. The COVID-19-related mortality is lower than the general population and vaccination for COVID-19 significantly reduces the risk of mortality.

## 1. Introduction

In December 2019, an outbreak of acute and highly lethal atypical pneumonia was observed in the Wuhan city of Hubei Province of China. It was later shown that this disease was a viral pneumonia caused by a novel coronavirus severe acute respiratory syndrome associated Corona Virus-2 (SARS-CoV-2). The disease was renamed as Coronavirus Infectious Disease-2019 (COVID-19) [1,2]. In January 2020, it was declared as Public Health Emergency of International Concern (PHEIC) which meant that the disease was a pandemic [1]. Waves of exacerbations were observed in the natural course of the pandemic. The clinical course of the infection varies among the individuals ranging from asymptomatic to mild and severe respiratory disease which increases the risk of mortality [3,4,5]. A major cause of death after COVID-19 infection is the cytokine storm and associated superinfection [3,4,5]. A special subgroup of patients was shown to have a higher risk of mortality following COVID-19. These include patients with older age, obesity, diabetes, hypertension, cardiovascular disease, chronic obstructive pulmonary disease, end stage renal disease, chronic liver disease, patients that require immunomodulatory medication (e.g., solid organ transplant recipients) and patients with malignancy [6,7,8]. Other factors associated with increased mortality include leukopenia, acute kidney injury and marked elevation of inflammatory markers [6,7,8].

In the initial stages of the pandemic, it was hypothesized that liver transplant (LT) recipients were at higher risk of mortality due to COVID-19 because of the immunosuppressive therapy and concomitant co-morbid diseases [9]. This hypothesis was not supported by majority of the studies as the reports showed that the course of COVID-19 infection in recipients of LT is similar to that of general population [10,11,12,13,14].

One of the concerns in LT recipients with COVID-19 is graft loss. There are two possible mechanisms for graft loss during COVID-19. First one is the direct cytopathic effect of the virus in hepatocytes due to upregulation of the ACE-2 receptors in the liver during regeneration, so the liver graft may especially be prone to infection with the virus [15,16]. The second mechanism is acute cellular rejection which results from the reduction in the dose of the immunosuppressive medication in the recipients during SARS-CoV-2 infection [17,18]. 

Our center has an annual LDLT volume of 250 to 300 cases. Furthermore, we have a recipient pool of more than 3000 individuals operated since the initiation of the transplant program in 2002. The main objective of this cohort study is to determine the demographic and clinical factors affecting mortality among LT recipients with COVID-19 infection.

## 2. Materials and Methods

### 2.1. Characteristics of the Study Cohort

Between March 2002 and November 2021, more than 3000 LT recipients underwent LT for various etiologies in our institute, but 1882 LT recipients who had sufficient data were analyzed for this retrospective cohort study. In addition, the data of the LT recipients who died during the COVID-19 period were also retrospectively analyzed.

### 2.2. Demographic Features and Data Collection

The following demographic and clinical characteristics were included in this cohort study: age, gender, height, weight, blood groups, marital status, occupation, level of education, comorbidity (diabetes mellitus, hypertension, pulmonary disease, etc.), smoking, immunosuppressive drugs (tacrolimus, everolimus, mycophenolate mofetil [MMF]), type of LT (LDLT, DDLT), retransplantation (reLT), diagnostic tools for COVID-19, lung involvement, antiviral drugs used for COVID-19, hospitalization, intensive care unit (ICU) stay, intubation status, COVID-19 vaccination, signs and symptoms related with COVID-19, awareness about COVID-19 and vaccination, white blood cell count (WBC), lymphocyte count, neutrophil count, platelet counts, aspartate aminotransferase (AST), alanine aminotransferase (ALT), total bilirubin (TBil), C-reactive protein (CRP), hemoglobin (Hb), mean platelet volume (MPV), alteration of the course of immunosuppressive medication (e.g., continue, reduced, interrupted) and patient outcomes (alive and dead). We have classified mortality as COVID-19-associated mortality which implies acute respiratory distress associated with the early phases of the disease (≤2 weeks). COVID-19 related mortality is defined as patient mortality related with cytokine storm and multiple organ failure which resulted in mortality in the later phase of the disease (>2 weeks).

### 2.3. Study Protocol and Ethics Committee Approval

This retrospective cohort study involving human participants was in accordance with the ethical standards of the institutional and national research committee and with the 1964 Helsinki Declaration and its later amendments or comparable ethical standards. First approval was obtained from the Ministry of Health of Turkey. Second approval was obtained from the Director of the Inonu University Liver Institute (Approval No: 2020/80462). Finally, ethics committee approval was obtained from the Inonu University institutional review board (IRB) for non-interventional clinical research (Approval No: 2020/1372).

### 2.4. Diagnosis of COVID-19 Infection

After the initial evaluation of physical signs and symptoms, the patients who were suspected to have COVID-19 were managed according to guidelines prepared by the Scientific Board of the Ministry of Health of Turkey. Reverse-transcription polymerase chain reaction (RT-PCR) test was performed by analyzing the nasopharyngeal and oropharyngeal swab samples. Thoracic computerized tomography (CT) was performed in LT recipients with negative RT-PCR tests but who were suspected to have COVID-19 pneumonia. Also, the thorax CT was performed in RT-PCR positive LT recipients to evaluate the extent of lung involvement in PCR positive patients. In patients with persistent clinical suspicion but negative PCR, the test was repeated 24–48 h after the previous test. The LT recipients diagnosed with COVID-19 were admitted to the clinics or intensive care units that were reserved for patients with COVID-19. These LT recipients were managed by multidisciplinary team according to the guidelines of the Ministry Health of Turkey. The data of the LT recipients treated in other centers were accessed both through the hospital information system and by telephone interview. We could not reach the laboratory values of 146 LT recipients that were treated elsewhere.

### 2.5. Statistical Analyses

Statistical analyses were performed using IBM SPSS Statistics for Windows version 26.0 (New York, NY, USA). Categorical variables were expressed as number of affected individuals and percentage of the study population. Pearson’s chi-square test, continuity-corrected chi-square test or Fisher’s exact test were used where appropriate for the comparison of categorical variables. Missing values for quantitative variables were assigned according to the median. Quantitative variables were expressed as median (interquartile range). The continuous variables in the two groups were compared with the Mann–Whitney U test. In this study, in addition to baseline comparisons, effect sizes (ES) were calculated to evaluate the effects of each variable in the patients who were alive and dead. ES is defined as the size of the difference between groups. The interpretation of Cramer’s V coefficient used in calculating the ES for chi-square tests is interpreted differently according to the minimum number of categories (k) in the rows and columns. If k = 2, 0.10–0.30 is interpreted as a small effect, 0.30–0.50 as a medium effect, and above 0.50 as a large effect. If k = 3, it is interpreted as a small effect between 0.07–0.20, a medium effect between 0.20–0.35, and a large effect over 0.35 [19]. For the Mann–Whitney U test, the ES (Cohen’s d) is interpreted as small effect between 0.20–0.50, medium effect between 0.50–0.80 and large effect above 0.80 [20]. Odds ratio (OR) estimates for variables with significant *p* values in chi-square tests were obtained by univariate logistic regression analysis. Variables with a significant *p* value in univariate analyzes were included in multivariate analysis using the backward method. Statistical tests with a *p* value of less than 5% were considered significant.

### 2.6. Data Preprocessing, Model Development and Feature Importance

A machine learning (ML) model was developed to predict all-cause mortality. At this stage, the LightGBM algorithm, which can process large-scale data with high accuracy and works based on histograms, was used [21]. Since the blood values of 241 of the 387 patients were measured, data of 241 patients were included in the model. Missing values were assigned according to the median. Standardization was applied to quantitative variables in the data. Of the 241 patients, 28 (11.6%) died and 213 (88.4%) lived. ML models tend to predict a certain class correctly in case of such a class imbalance problem. For this reason, the class imbalance problem has been fixed with Synthetic Minority Over-sampling Technique (SMOTE). By learning from the original data, the sample numbers in the groups were equalized with SMOTE [22].

**Figure 1:**
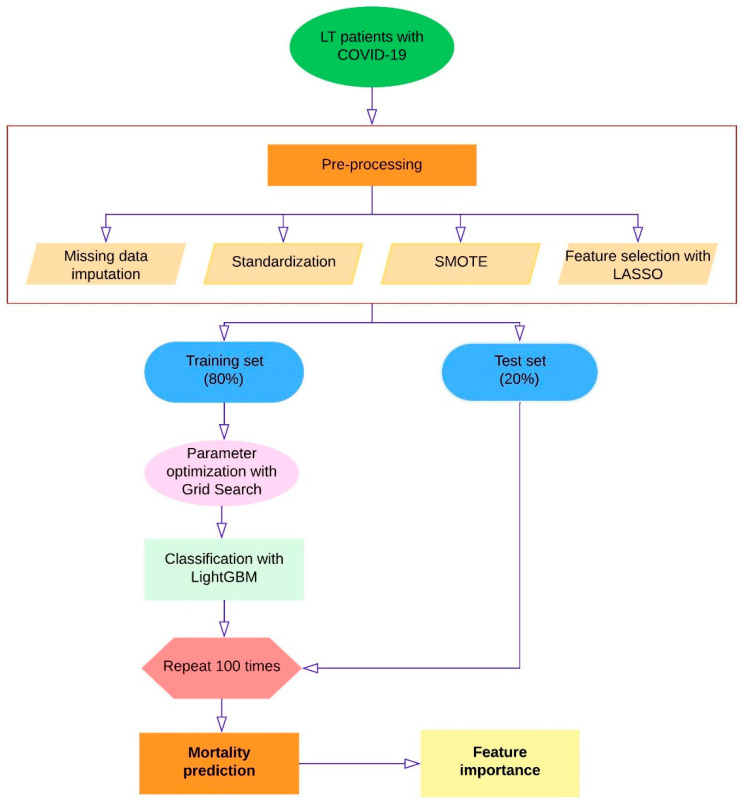
Diagram of the methodology for ML estimation of mortality in LT patients with COVID-19.

Stratified random sampling method was used to divide the obtained 426 samples into training and test sets at a ratio of 4:1. LASSO [23] feature selection method was used to determine the biomarkers that had the greatest impact on mortality. The hyper-parameters of the LightGBM model were optimized using a random search method with tenfold cross-validation. Finally, the performance of the model was evaluated on the test set. To obtain more robust performance on the test set and report results unbiasedly, we repeated the validation method 100 times with different random seeds and calculated the average scores for these measurements for each performance measurement (Figure 1). The model, which was created with multiple evaluation criteria such as accuracy, f1 score, precision, recall and AUC, was comprehensively evaluated. Finally, the order of importance of the variables contributing to the model for mortality risk estimation and the feature importance graph were given and the model was explained clinically.

## 3. Results

### 3.1. General Characteristics of the Study Population

Between March 2002 and November 2021, we performed about 3000 LT for various indications. Only 387 patients developed COVID-19 infections during the follow and they were included in the study.

All the 387 LT recipients who were diagnosed with COVID-19 were included in the study. The median age of the patients was 53 (IQR: 20, min-max: 1–72 years) years. In total, 111 (28.7%) patients were female and 276 (71.3%) were male. During the follow up period, 28 patients died due to COVID-19 infection. Of these, 14 of the patients died directly from the COVID-19 infection while the remaining 14 died from COVID-19 related complications. The median duration of survival of the patients who died from COVID-19 infection was 15.5 days (IQR: 20). The median survival period of the patients who died from COVID-19 related complications was 30.5 (IQR: 51) days. The median (95% CI) duration from LT procedure to COVID-19 of the patients who were on prednisolone was 10 months (6–20 months). On the other hand, the median (95% CI) interval between LT procedure and COVID-19 of the patients who are not on prednisolone was 60 months (53–72 months). The demographic and clinical characteristics of the LT recipients with COVID-19 are summarized in Table 1. The COVID-19-related data of the LT recipients are summarized in Table 2.

### 3.2. The Factors Determining Survival and Mortality

To analyze the risk factors affecting mortality, the LT recipients with COVID-19 infection were divided into two subgroups; patients who survived (*n* = 359) and the patients that died from the COVID-19 infection (*n* = 28). Comparison of quantitative variables of the LT recipients is summarized in Table 3. There is no difference in terms of mortality among pediatric and adult patients (*p* = 0.085). Among the 28 mortalities observed, 4 were in pediatric patients and 24 adult patients, respectively. We observed an inverse relationship between the patients’ weight and mortality from COVID-19 infection with a *p*-value of 0.003. However, the ES showed that this difference among the patients’ weight was not clinically significant (Cohen’s d: 0.3). Other demographic parameters such as age (*p* = 0.168), height (*p* = 0.447), number of COVID-19 episodes (*p* = 0.413) and the duration between the initiation of symptoms and the diagnosis (*p* = 0.976) did not show significant differences between the two group of LT recipients.

A comparison of qualitative variables of the LT recipients is summarized in Table 4. We found a significant difference between the two groups when we considered reLT (*p* = 0.027), tacrolimus therapy (*p* = 0.015), everolimus therapy (*p* = 0.009) and prednisolone administration (*p* = 0.048). The ES value of reLT (Cramér’s V = 0.135), tacrolimus therapy (Cramér’s V = 0.138), everolimus therapy (Cramér’s V = 0.143) and prednisolone administration (Cramér’s V = 0.109) indicated that these parameters had some impact on the mortality of the patients. The results of the univariate analysis showed that StatreLT increased the mortality risk by 4.4 fold (95% CI = 1.3–14.6), everolimus therapy increased the mortality risk by 2.94 fold (95% CI = 1.35–6.42) and prednisolone administration increased the risk of mortality by 2.53 fold (95% CI = 1.01–6.06). On the other hand, tacrolimus therapy reduced the risk of mortality by 3.44 fold (95% CI = 1.35–8.33).

Furthermore, we have analyzed the LT cohort in terms of the interval between the LT procedure until contracting COVID-19. We have grouped the patients into two groups according to the time interval since LT (≤1 year versus 1 year) and no statistically significant difference was found between the groups in terms of mortality rates (*p* = 0.102). In total, 8 patients who died due to COVID-19-related etiologies received a liver transplant ≤1 year; on the other hand, 20 patients who died related to COVID-19 etiologies received a liver transplant >1 year. As we have stated previously, a total of 304 patients received vaccination. There is no difference in mortality among patients who did and did not contract COVID-19 following vaccination (*p* = 1.0). However, the number of patients is low. A more striking result is that 24 patients who died due to COVID-19 related etiologies have expired before the era of vaccination.

We have tried to analyze the severity of inflammation among our patients by analyzing the Procalcitonin and C-reactive Protein levels of the patients and we not found a significant difference in terms of procalcitonin (*p* = 0.181) and C-reactive protein (*p* = 0.574) levels among the patients with and without mortality.

A comparison of qualitative variables regarding the COVID-19-related symptoms is summarized in Table 5. We found a significant difference between the two groups of patients when we compared lung involvement (*p* = 0.002), antiviral drugs use for COVID-19 (*p* = 0.027), hospitalization (*p* < 0.001), ICU stay (*p* < 0.001), need for intubation (*p* < 0.001), COVID-19 vaccination (*p* < 0.001), shortness of breath (*p* = 0.03) and alteration of immunosuppressive drugs during COVID-19 (*p* < 0.001). When we calculated the ES value, we found that the values for lung involvement (Cramér’s V = 0.169), antiviral drugs use (Cramér’s V = 0.123), hospitalization (Cramér’s V = 0.27) and shortness of breath (Cramér’s V = 0.122) were very small. On the other hand, the ES values for COVID-19 vaccination (Cramér’s V = 0.437) and alteration of immunosuppressive drugs during COVID-19 (Cramér’s V = 0.397) were moderately high ES values. This shows that COVID-19 vaccination and alteration of the immunosuppressive medications had higher clinical importance. ICU stay (Cramér’s V = 0.599) and need for intubation (Cramér’s V = 0.714) had the highest ES values and had the highest clinical significance. The univariate analyses of the variables in Table 5 showed that lung involvement increased the risk of mortality by 3.5 fold [95% CI = 1.6–7.7], the use of antiviral drugs increased the mortality risk by 2.67 fold [95% CI = 1.2–6.0], the need for hospitalization increased the risk of mortality by 8.8 fold [95% CI = 3.4–22] and the need for ICU stay increased the mortality risk by 59 fold [95% CI = 22–161]. However, the need for intubation increased the risk of mortality by 149.5 fold [95% CI = 46–490]. On the other hand, the patients with dyspnea had an increased mortality risk of 2.5 fold [95% CI = 1.2–5.5]. Reduction of the doses of the immunosuppressive medications increased the mortality risk by 13.8 fold [95% CI = 3.6–52.1]. However, discontinuation of medication increased the mortality risk by 15.7 fold [95% CI = 6.3–39.2]. Immunization before contracting COVID-19 reduced the risk of mortality by 33.3 fold [95% CI = 11–100].

Table 6 summarizes the data related to COVID-19 vaccination. In total, 304 (78.6%) LT recipients were vaccinated. The most preferred COVID-19 vaccine was BioNTech in 139 LT recipients (45.7%), followed by Sinovac in 101 (33.2%). Sixty-four (21.1%) patients had been vaccinated by both type of vaccines. A total of 100 LT recipients were diagnosed with COVID-19 at least one time despite vaccination and 68 (68%) of these patients received Sinovac while 25 (25%) received BioNtech vaccines. The LT recipients who were infected following vaccination had at least two doses of vaccines in 88% of the cases.

Table 7 summarizes the opinions of the LT recipients with COVID-19 before (group 1) and after vaccination (group 2) regarding COVID-19 vaccination. The concerns about vaccination differed significantly among the two groups (*p* = 0.018; OR = 1.48). However, ES showed that the difference could be negligible (Cramér’s V = 0.146). There was no difference in terms of variables such as opinions regarding the protective capacity of the vaccine (*p* = 0.500), and the views of the LT recipients on the vaccine being legally obligatory (*p* = 0.500).

Table 8 summarizes the quantitative variables of the LT recipients of patients in both groups. We found a statistically significant difference between the two groups when we compared variables such as lymphocyte (*p* < 0.001), platelets (*p* = 0.013), Tbil (*p* < 0.001), Hb (*p* < 0.001) and MPV (*p* = 0.012). When we calculated the ES, we found that platelets (Cohen’s d = 0.323) and MPV (Cohen’s d = 0.328) had moderate or low ES values which precludes its clinical significance in terms of mortality. On the other hand, lymphocyte (Cohen’s d = 0.577) and Hb (Cohen’s d = 0.604) had higher ES values which indicates their importance in predicting mortality in clinical practice. TBil (Cohen’s d = 0.853) had the highest ES values which indicates that it has the highest impact in predicting mortality. Lymphocyte (decreased; OR = 2.1) and platelets (decreased; OR = 1.01), Tbil (increased; OR = 1.40), Hb; OR = 1.00) and MPV (increased; OR = 1.03) had significant correlations with the risk of mortality.

### 3.3. The Independent Risk Factors of Mortality

Table 9 summarizes the results of multivariate analysis of the impact of variables on mortality in LT recipients. Everolimus therapy (*p* = 0.012; OR = 6.2), need for intubation (*p* = 0.001; OR = 38.4) and discontinuation of immunosuppressive drugs (*p* = 0.047; OR = 7.3) were significant and independent risk factors predicting mortality. Furthermore, COVID-19 vaccination reduced the risk of mortality by 100 fold and was the single independent factor determining the survival of the LT recipients.

**Figure 2:**
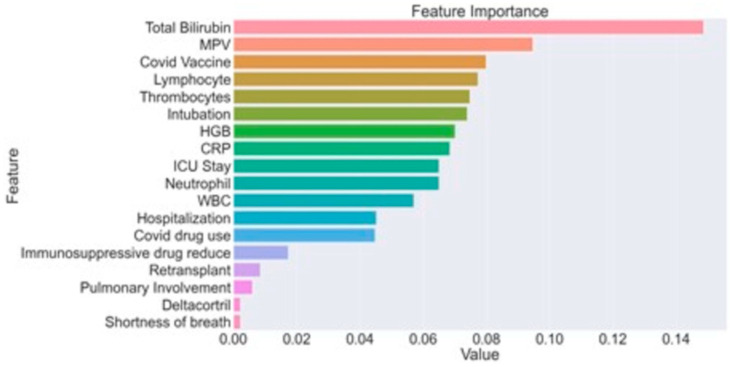
Significance plot for mortality prediction of various variables.

Table 10 performance measures and mortality prediction models obtained from the LightGBM model. LightGBM model predicted the mortality of the LT recipients with a 96.64% accuracy. In addition, the f1 score, precision, recall and AUC parameters calculated from the model were 96.6%, 97.7%, 96.1% and 99.6%, respectively. Table 11 summarizes mortality prediction performance of 18 parameters obtained from LASSO model. Figure 2 summarizes the importance of 18 variables in prediction of mortality. Figure 2 and Table 10 summarize the significant factors that have the highest contribution in predicting the mortality in the LT recipients. The most prominent factors were Tbil (14.9%), MPV (9.5%), COVID-19 vaccination (8.0%), lymphocyte (7.7%), platelets (7.5%) and the need for intubation (7.4%).

## 4. Discussion

This study is unique because it is the only single-center, high-volume study evaluating the clinical features of COVID-19 in LT recipients. Furthermore, we evaluated multiple aspects of the disease including clinical characteristics, laboratory values, effect of immunosuppression therapy and vaccination in this highly specialized cohort.

The outcomes of COVID-19 in LT recipients are not uniform. While some studies have reported increased mortality, other studies are reporting comparable or decreased mortality in comparison to the general population [9,10,11,13,24]. In our study, the mortality rate of COVID-19 among our patients is 7.2%. Reports from 2020 and 2021 tend to show higher mortality compared to reports after 2021. Studies in the literature have reported a mortality rate ranging between 4 and 20% varying according to the duration that the study was performed [10,11,14,25,26]. Contrary to the expectations, the mortality rate among the LT recipients is low. The mortality is mainly due to the exacerbated immune response and the resultant cytokine storm. The immuno-suppressive medications may have suppressed this response and reduced the mortality among recipients of organ transplant [27,28,29]. Our study is the proof of concept for this theory. In our study, the use of tacrolimus was associated with reduced mortality. In addition, tapering immunosuppressive therapy increased the risk of mortality. However, we did not observe the same result with the use of everolimus and steroids. The difference may be related to the findings that tacrolimus is superior to everolimus in inhibiting cellular alloimmunity [30]. There have been previous reports showing no significant effect of immunosuppressive and immunomodulatory medication in increasing the risk of severe COVID-19 in adults with autoimmune diseases or children with various rheumatic/autoimmune kidney disease [31,32]. Furthermore, Marlais et al. [33] have analyzed the impact of immunosuppressives on 582 pediatric patients with renal disease (53 of which were renal transplant recipients) and observed no increased risk for developing severe COVID-19. The present study is the first in the literature to emphasize the results of immunosuppressive therapy in LT recipients who have COVID-19. The results of this study should be evaluated with caution because the immunosuppressive regimen is very heterogeneous in LT recipients. Generally, the backbone of the immunosuppression is calcineurin inhibitors which are coupled with prednisolone and or antimetabolites such as MMF. In the present study, the patients receiving prednisolone are in the early post-transplant period (median 10 months). Our institute usually weans from steroids between the 3rd to 6th postoperative months unless the patient has an autoimmune liver disorder. Furthermore, the SARS-CoV-2 variants responsible for different waves have various mortality rates which is also unknown in the present study. Nevertheless, this also may have resulted in variations in the mortality of the LT recipients.

There are studies evaluating the effects of different immunosuppressives on the immunogenicity of mRNA-based vaccines for SARS-CoV-2 [34,35,36,37]. All the studies have shown that MMF reduced the efficacy of the vaccine in liver transplant recipients. The results regarding steroids have not shown any significant effect. Our study evaluated the impact of vaccination on the outcome of liver transplant recipients, and we have found that tacrolimus reduced the risk of mortality in these patients. We could not find a significant relationship between other different immunosuppressive regimens and mortality risk of the patients.

Lymphopenia was reported to be an independent risk factor in predicting mortality in patients with COVID-19 [38,39]. In our study, we notice that lower Hb, platelet, and lymphocyte count is associated with increased mortality. We also found that the use of everolimus increased the mortality risk in our patients. This is mainly due to its myelosuppressive effects [40]. Furthermore, patients who received re-transplantation had higher mortality following COVID-19. Although there are no data in the literature regarding the immune consequences of liver re-transplantation, the need for the use of combination immune suppressants and the need for anti-plasma cell therapy may exacerbate the myelosuppression causing an increased risk of mortality due to COVID-19.

Bilirubin has long been defined as a prognostic factor in patients with acute or chronic end-stage liver disease [41,42,43,44]. It is also a component of the model for the end-stage liver disease (MELD) scoring system [45]. In addition, liver injury during COVID-19 has been implicated in various studies, and universally, all researchers have stated abnormal liver function tests as a prognostic marker in COVID-19 [46,47,48]. However, the effects of COVID-19 in a transplanted liver graft have not been shown. In our study, we found that in both multivariate analyses and AI prediction models, bilirubin is an independent predictor of mortality among our patients. Our results indicate two important issues. First is that bilirubin is an accurate marker that predicts the function of the liver graft during physiologic stress. The second is the fact that cytokine storm, direct viral replication and the effect of medical therapy used during the infection in patients with COVID-19 causes a certain level of liver injury and, occasionally, may cause graft failure and loss of the patients.

Platelets have important functions in liver regeneration, and also the ischemia-reperfusion injury observed during LT and has been studied extensively in partial liver grafts [49,50,51]. In this study, we found that lower platelets count is a predictor of mortality among our patients. This may be due to the unrestrained systemic inflammatory process seen in COVID-19. During unrestrained systemic inflammation, thrombocytopenia may develop due to reduced production in the bone marrow, hemodilution, consumption due to disseminated intravascular coagulation, sequestration in the microvasculature and destruction by immune-mediated mechanisms [52].

Admission into the intensive care unit and intubation are associated with increased risk of mortality in patients with COVID-19 [14,38,53,54]. Furthermore, old age, male gender and obesity were the most reported risk factors for mortality among solid organ transplant recipients [55]. Similarly, in this study, we found that the need for intubation and ICU admission were independent risk factors for mortality with high clinical significance. Furthermore, dyspnea increased the risk of mortality although the ES showed that this association was weak. Nevertheless, our results show that LT recipients are not completely different from the general population in terms of risk factors for poor outcome and clinical features of COVID-19.

The COVID-19 vaccines reduce the mortality of COVID-19 in solid organ transplant recipients by 2 to 8 fold [56,57]. Furthermore, it has been shown that commercially available vaccines in Europe have increased the protection from the infection from 32% (in a single dose) to 72% (in a triple dose) [58]. Our study is especially important because we have both mRNA vaccines as well as inactivated vaccines in Turkey. Also, the vaccination rate in our patient cohort was 78%. Among the study population, 45% of the patients received mRNA vaccine and 33% received inactivated vaccines. The vaccines reduced the mortality by 100 fold in our study. In our study, we also found that the type of vaccine has no effect on the protection offered by the COVID vaccine. The most prominent point that should be emphasized is that 63% of our patients received at least two doses of vaccines and 85% received at least three doses. Therefore, our results show that inactivated vaccines are effective as mRNA vaccines, and also at least two doses are enough for an effective protection. In future we would like to compare the results of our patients with healthy population to evaluate the effect of immunosuppression on the efficacy of the vaccines.

One of the most important aspects of the current study is the use of artificial intelligence (AI) for development of a prognostic model for COVID-19 in LT recipients. The AI model determined total bilirubin, MPV, COVID-19 vaccination status, need for intubation, lymphocyte and platelet counts as potential factors determining the prognosis of the LT recipients with COVID-19. Machine learning in medical research enables evaluation of mass data and evaluation of complex medical problems where data is limited. It is an algorithm-based system that develops a predictable pattern in the guidance of the data regarding the research subject [59]. Therefore, machine learning is suitable for a complex entity such as COVID-19 in a highly susceptible subgroup of patients.

The present study is the first single center high volume study evaluating the outcome of COVID-19 in solid organ transplant recipients. The results are striking and delineates independent predictors of mortality among liver transplant recipients with COVID 19 infection, the efficacy of the vaccines in protection against COVID infection among recipients and we present a predictive model for determining the mortality in the patients. However, the present study has some limitations. The retrospective nature of the study precluded reaching necessary data in some of the patients. Furthermore, some of the recipients who had COVID-19 were diagnosed and treated in other centers and the laboratory parameters showed differences.

## 5. Limitations

The present study is designed as a retrospective cohort study. The results of retrospective studies are not as powerful as prospective cohort studies. Furthermore, we could not perform a matched cohort of non-transplanted patients which is another limitation. We could not select a matched cohort because data regarding the course of COVID-19 in the Turkish population is lacking. However, it is impossible to make prospective studies during a pandemic. The present study includes the data of our patients in the first two years of the pandemic. The patients in the last year of the study were not included, which is a very important limitation of our study. The majority of the patients lived in different parts of Turkey and in the beginning of the pandemic, the transportation between the centers were severely limited. For this reason, our patients with COVID-19 had to be treated in their cities of residence. The clinical approach of different physicians treating these patients severely limited the laboratory values obtained. Furthermore, the majority of the physicians struggling with the pandemic focused primarily on the clinical situation of the patients rather than the academic dimension of this catastrophe. Hence, we could not obtain many laboratory parameters for our patients and the parameters that were obtained were not uniform. Despite all these limitations, our study included a large volume of patients which we believe should increase the power of our results.

In conclusion, LT recipients do not have an elevated risk of mortality related to COVID-19. Furthermore, risk factors for mortality are similar to non-transplanted population. Vaccines are extremely effective in reducing COVID-19-related mortality in solid organ transplant recipients. Globally, governments should take precautions to promote vaccination and to reduce vaccine opposition.

## Figures and Tables

**Table 1:** Demographic and clinical characteristics of LT recipients who had COVID-19.

Qualitative Variables	Categories	*n* (%)
Gender	Female	111 (28.7)
	Male	276 (71.3)
Blood groups	A	170 (44.6)
	B	64 (16.8)
	AB	36 (9.5)
	0	111 (29.1)
Marital status	Single	64 (16.5)
	Married	316 (81.7)
	Divorced	7 (1.8)
Occupation (job)	Retired	155 (40.2)
	Unemployed	188 (48.7)
	Employed	43 (11.1)
Educational level	Illiterate	78 (20.2)
	Primary school	165 (42.6)
	Secondary school	48 (12.4)
	High school	57 (14.7)
	Bachelor’s degree or more	39 (10.1)
Diabetes mellitus	Yes	109 (28.2)
	No	278 (71.8)
Hypertension	Yes	68 (17.57)
	No	319 (82.4)
Pulmonary disease	Yes	12 (3.1)
	No	375 (96.9)
Smoking	Yes	51 (13.2)
	No	336 (86.8)
Tacrolimus therapy	Yes	348 (89.9)
	No	39 (10.1)
Everolimus therapy	Yes	128 (33.1)
	No	259 (66.9)
MMF therapy	Yes	35 (9.0)
	No	352 (90.9)
Prednisolone therapy	Yes	57 (14.7)
	No	330 (85.3)
Type of LT	LDLT	348 (89.9)
	DDLT	39 (10.1)
Need for reLT	Yes	17 (4.4)
	No	370 (95.6)

MMF: Mycophenolate mofetil; reLT: re-liver transplantation.

**Table 2:** Descriptive statistics related to COVID-19 in LT recipients.

Qualitative Variables	Categories	*n* (%)
Diagnostic tools for COVID-19	PCR	369 (95.4)
	CT	18 (4.7)
Lung involvement	Yes	93 (24.1)
	No	293 (75.9)
Need for antiviral drugs to treat COVID-19	Yes	178 (46.0)
	No	209 (54.0)
Hospitalization	Yes	128 (33.1)
	No	259 (66.9)
ICU stay	Yes	43 (11.1)
	No	344 (88.9)
History of intubation	Yes	24 (6.2)
	No	363 (93.8)
COVID-19 vaccine administration	Yes	304 (78.6)
	No	83 (21.5)
Hesitancy against COVID-19 vaccine	Yes	62 (17.4)
	No	294 (82.6)
Belief in the efficacy of COVID-19 vaccines	Yes	308 (87.5)
	No	44 (12.5)
Approval of legally mandating COVID-19 vaccination	Yes	233 (67.7)
	No	111 (32.3)
Fever	Yes	178 (46.11)
	No	208 (53.9)
Anorexia	Yes	155 (40.5)
	No	228 (59.5)
Cough	Yes	172 (44.6)
	No	214 (55.4)
Shortness of breath	Yes	116 (30.1)
	No	270 (70.0)
Headache	Yes	169 (43.8)
	No	217 (56.2)
Backache	Yes	146 (37.8)
	No	240 (62.18)
Diarrhea	Yes	81 (21.1)
	No	303 (78.9)
Fatigue/exhaustion	Yes	233 (60.4)
	No	153 (39.6)
Muscle joint pain	Yes	210 (54.4)
	No	176 (45.6)
Loss of taste or smell	Yes	121 (31.4)
	No	264 (68.6)

**Table 3:** Comparison of quantitative variables of LT recipients according to survival status.

Quantitative Variables	Outcome				*p*	EF
	Alive (*n* = 359)		Dead (*n* = 28)			
	Median (IQR)	95% CI	Median (IQR)	95% CI		
Age	53 (20)	[51–55]	56.5 (21.5)	[53–64]	0.168 *	0.3
Height	169 (13)	[168–170]	166 (11.5)	[166–172]	0.447 *	
Weight	75 (18)	[74–78]	67.5 (15.9)	[60–72]	0.003 *	
Doses of COVID-19 vaccination	1(0)		1(0)		0.413 *	
Interval between the initiation of symptom to COVID-19 diagnosis (days)	2(2)	[2,3]	2(1)	[2,3]	0.976 *	

*: Mann–Whitney U test, EF: effect size.

**Table 4:** Comparison of qualitative variables of LT recipients according to outcome of the patients.

Qualitative Variables (*n* [%])		Outcome		*p*	ES	OR [95% CI]
		Alive (*n* = 359)	Dead (*n* = 28)			
Type of LT	LDLT	325 (90.5)	23 (82.1)	0.180 *		
	DDLT	34 (9.5)	5 (17.9)			
Need for reLT	Yes	13 (3.6)	4 (14.3)	0.027 *	0.135	4.4 [1.3–14.6]
	No	346 (96.4)	24 (85.7)			
Gender	Female	106 (29.5)	5 (17.9)	0.270 **		
	Male	253 (70.5)	23 (82.1)			
Blood groups	A	153 (43.3)	17 (60.7)	0.290 ***		
	B	61 (17.3)	3 (10.7)			
	AB	35 (9.9)	1 (3.6)			
	0	104 (29.5)	7 (25)			
Marital Status	Single	56 (15.6)	8 (28.6)	0.160 ***		
	Married	296 (82.4)	20 (71.4)			
	Divorced	7 (2.0)	0 (0)			
Occupation (job)	Retired	140 (39.0)	15 (55.6)	0.080 ***		
	Unemployed	176 (49.0)	12 (44.4)			
	Employed	43 (12.0)	0 (0)			
Diabetes mellitus	Yes	101 (28.1)	8 (28.6	1.000 **		
	No	258 (71.9)	20 (71.4)			
Hypertension	Yes	60 (16.7)	8 (28.6)	0.120 *		
	No	299 (83.3)	20 (71.4)			
Pulmonary disease	Yes	11 (3.1)	1 (3.6)	0.590 *		
	No	348 (96.9)	27 (96.4			
Smoking	Yes	47 (13.1)	4 (14.3)	0.770 *		
	No	312 (86.9)	24 (85.71)			
Tacrolimus therapy	Yes	327 (91.1)	21 (75)	0.015 *	0.138	0.29 [0.12–0.74]
	No	32 (8.9)	7 (25)			
Everolimus therapy	Yes	112 (31.2)	16 (57.14)	0.009 **	0.143	2.94 [1.35–6.42]
	No	247 (68.8)	12 (42.9)			
MMF therapy	Yes	31 (8.6)	4 (14.3)	0.300 *		
	No	328 (91.4)	24 (85.7)			
Prednisolone therapy	Yes	49 (13.6)	8 (28.6)	0.048 *	0.109	2.53 [1.01–6.16]
	No	310 (86.4)	20(71.4)			

*: Fisher’s exact test; **: Yates continuity correction test; ***: Pearson’s chi-squared test; ES: effect size; MMF: mycophenolate mofetil; reLT: re-liver transplantation.

**Table 5:** Comparison of qualitative variables of LT recipients according to survival status.

Qualitative Variables (*n* [%])		Outcome		*p*	ES	OR [95% CI]
		Alive	Dead			
Diagnostic tools for COVID-19	PCR	342 (95.3)	27 (96.4)	1 ***		
	CT	17 (4.7)	1 (3.6)			
Lung involvement	Yes	79 (22.1)	14 (50)	0.002 ***	0.169	3.5 [1.6–7.7]
	No	279 (77.9)	14 (50)			
Need for antiviral drugs to treat COVID-19	Yes	159 (44.3)	19 (67.86)	0.027 **	0.123	2.67 [1.2–6]
	No	200 (55.7)	9 (32.14)			
Hospitalization	Yes	106 (29.5)	22 (78.6)	<0.001 **	0.27	8.8 [3.4–22]
	No	253 (70.5)	6 (21.4)			
ICU stay	Yes	21 (5.8)	22 (78.6)	<0.001 ***	0.599	59.0 [22–161]
	No	338 (94.2)	6 (21.4)			
History of intubation	Yes	5 (1.4)	19 (67.9)	<0.001 ***	0.714	149.5 [46–490]
	No	354 (98.6)	9 (32.1)			
COVID-19 vaccine administration	Yes	300 (83.6)	4 (14.3)	<0.001 **	0.437	0.03 [0.01–0.09]
	No	59 (16.4)	24 (85.7)			
Hesitancy against COVID-19 vaccine	Yes	62 (17.6)	0 (0)	1 ***		
	No	290 (82.4)	4 (100)			
Belief in the efficacy of COVID-19 vaccines	Yes	305 (87.4)	3 (100)	1 ***		
	No	44 (12.6)	0 (0)			
Approval of legally mandating COVID-19 vaccination	Yes	231 (67.7)	2 (66.7)	1 ***		
	No	110 (32.3)	1 (33.3)			
Fever	Yes	164 (45.8)	14 (50)	0.817 **		
	No	194 (54.2)	14 (50)			
Anorexia	Yes	141 (39.5)	14 (53.8)	0.218 **		
	No	216 (60.5)	12 (46.2)			
Cough	Yes	158 (44.1)	14 (50)	0.686 **		
	No	200 (55.9)	14 (50)			
Shortness of breath	Yes	102 (28.5)	14 (50)	0.03 **	0.122	2.5 [1.2–5.5]
	No	256 (71.5)	14 (50)			
Headache	Yes	158 (44.1)	11 (39.3)	0.764 **		
	No	200 (55.9)	17 (60.7)			
Backache	Yes	135 (37.7)	11 (39.3)	1 **		
	No	223 (62.3)	17 (60.7)			
Diarrhea	Yes	76 (21.4)	5 (17.9)	0.845 **		
	No	280 (78.6)	23 (82.1)			
Fatigue/exhaustion	Yes	216 (60.3)	17 (60.7)	1 **		
	No	142 (39.7)	11 (39.3)			
Muscle joint pain	Yes	193 (53.9)	17 (60.7)	0.618 **		
	No	165 (46.1)	11 (39.3)			
Loss of taste	Yes	116 (32.5)	5 (17.9)	0.163 **		
	No	241 (67.5)	23 (82.1)			
Loss of smell	Yes	107 (30.1)	4 (14.3)	0.12 **		
	No	249 (69.9)	24 (85.7)			
Immunosuppressive drugs during COVID-19	Continued	330 ^a^ (91.9)	12 ^b^ (42.9)	<0.001 *	0.397	
	Reduced	8 ^a^ (2.2)	4 ^b^ (14.3)			13.8 [3.6–52.1]
	Interrupted	21 ^a^ (5.9)	12 ^b^ (42.6)			15.7 [6.3–39.2]

*: Pearson ki-kare test; **: Yates Continuity Correction test; ***: Fisher’s exact test; a, b: different letters in each row show a statistical significant difference (*p* < 0.05).

**Table 6:** Descriptive statistics related to COVID-19 vaccination.

Qualitative Variables		*n* (%)
COVID-19 vaccine administration	Yes	304 (78.6)
	No	83 (21.4)
Type of COVID-19 vaccine	Sinovac™	101 (33.2)
	BioNTech™	139 (45.7)
	Both	64 (21.1)
Number of COVID-19 vaccine doses	One	20 (6.6)
	Two	126 (41.4)
	Three	119 (39.1)
	Four	39 (12.8)
Exposed to COVID-19 after vaccination	Yes	100 (32.9)
	No	204 (67.1)
Vaccine had been administered to patients diagnosed with COVID-19	Sinovac™	68 (68)
	Biontech™	25 (25)
	Both	7 (7)
Vaccine doses in patients before COVID-19 diagnosis	First	12 (12)
	Second	63 (63)
	Third	22 (22)
	Fourth	3 (3)

**Table 7:** The impact of vaccination administered before or after diagnosis of COVID-19 on perception towards vaccines.

Qualitative Variables (n [%])		Exposure to COVID-19		*p*	ES	OR [95% CI]
		after Vaccination	before Vaccination			
Hesitancy against COVID-19 vaccine	Yes	19 (19)	18 (8.8)	0.018 *	0.146	0.68 [0.07–6.59]
	No	81 (81)	186 (91.2)			
Belief in the efficacy of COVID-19 vaccines	Yes	88 (90.7)	191 (93.6)	0.5 *		
	No	9 (9.3)	13 (6.4)			
Approval of legally mandating COVID-19 vaccination	Yes	73 (76)	147 (75.4)	1 *		
	No	23 (24)	48 (24.6)			

*: Yates Continuity Correction test.

**Table 8:** Comparison of laboratory values of LT recipients according to outcome of the patients.

Quantitative Variables	Outcome				*p*	ES	OR [95% CI]
	Alive (*n* = 213)		Dead (*n* = 28)				
	Median	95% CI	Median	95% CI			
WBC	5.3 (3.7)	[5,6]	6.15 (0.35)	[6.15–7]	0.309		
Lymphocyte	1.1 (1.1)	[1–1.3]	0.45 (0.3)	[0.45–1.6]	<0.001 *	0.577	0.48 [0.25–0.88] *
Neutrophil	3.3 (2.7)	[3,4]	4.15 (1.105)	[4.15–5.5]	0.165		
Platelets	160 (102)	[151–175]	141 (24.5)	[141–164]	0.013 *	0.323	0.99 [0.99–0.99] *
AST	28 (22)	[27–32]	30 (15)	[30–63]	0.788		
ALT	25 (25)	[24–30]	23 (13)	[23–76]	0.611		
Total bilirubin	0.6 (0.4)	[0.6–0.8]	2.75 (2.4)	[2.75–8.2]	<0.001 *	0.853	1.40 [1.18–1.66] *
CRP	3.6 (6.5)	[3.6–5.2]	3.7 (0.3)	[3.7–7.3]	0.574		
Hb	12.55 (4)	[12–13.1]	9.7 (0.9)	[9.7–12]	<0.001 *	0.604	1.00 [0.98–1.03]
MPV	10 (1.7)	[10–10.5]	10.3 (0)	[10.3–11]	0.012 *	0.328	1.03 [0.86–1.23]

*: Statistically Significant.

**Table 9:** The factors related with mortality.

Variables in the Equation (*n* = 387)	OR	95% CI	*p*
Everolimus therapy	6.2	[1.51–25.4]	0.012
Hospitalization	0.05	[0.001–2.03]	0.111
ICU stay	36.2	[0.89–1464.6]	0.057
Intubation	38.4	[4.14–356.71]	0.001
COVID-19 vaccination	0.01	[0.002–0.10]	<0.001
Reduction of immunosuppressive drugs	0.5	[0.025–10.80]	0.672
Interruption of immunosuppressive drugs	7.3	[1.03–51.25]	0.047

OR: Odds ratio, CI: Confidence Interval.

**Table 10:** The ML model for prediction of mortality in LT recipients with COVID-19.

Model	Accuracy	F1 Score	Precision	Recall	AUC
LightGBM	0.9694 ± 0.04	0.966 ± 0.03	0.977 ± 0.04	0.961 ± 0.06	0.996 ± 0.01

AUC: Area under the ROC Curve.

**Table 11:** The impact of variables on the mortality using ML model for prediction.

Features	Value
Total Bilirubin	0.149
MPV	0.095
COVID-19 vaccination	0.080
Lymphocyte	0.077
Platelets	0.075
Intubation	0.074
Hb	0.070
CRP	0.068
ICU stay	0.065
Neutrophil	0.065
WBC	0.057
Hospitalization	0.045
Antiviral drugs for COVID-19	0.045
Alteration of the course of immunosuppressive medications	0.017
Need for reLT	0.008
Lung involvement	0.006
Prednisolone administration	0.002
Shortness of breath	0.002

reLT: reliver transplantation; ICU: intensive care unit; CRP: c-reactive protein.

## Data Availability

The datasets analyzed during the current study are available from the corresponding author on reasonable request.
